# Novel mutations in the *RECQL4* gene affect its helicase functions, interactions with the BLM helicase and chemotherapeutics-induced cell death

**DOI:** 10.1038/s41420-025-02834-w

**Published:** 2025-12-19

**Authors:** Agnieszka Kaczmarczyk, Mikolaj Sokolowski, Kamil Wojnicki, Marta Pabis, Bartosz Wojtas, Iwona A. Ciechomska, Katarzyna Poleszak, Bartlomiej Gielniewski, Sylwia K. Król, Matthew Guille, Sebastian Glatt, Bozena Kaminska

**Affiliations:** 1 Laboratory of Molecular Neurobiology, Nencki Institute of Experimental Biology of the Polish Academy of Sciences, Warsaw, Poland; 2 Malopolska Centre of Biotechnology, Jagiellonian University, Kraków, Poland; 3 Postgraduate School of Molecular Medicine, Warsaw, Poland; 4 Laboratory of Sequencing, Nencki Institute of Experimental Biology of the Polish Academy of Sciences, Warsaw, Poland; 5 European Xenopus Resource, School of Biological Sciences, University of Portsmouth, Portsmouth, PO1 2UP United Kingdom; 6 Department for Biological Sciences and Pathobiology, University of Veterinary Medicine Vienna, 1210 Vienna, Austria

**Keywords:** DNA damage and repair, Cancer genomics, Molecular modelling

## Abstract

RecQ family of DNA helicases play pivotal roles in DNA replication, repair and responses to DNA damage or replication stress. Several human RecQ helicases are defective in diseases associated with chromosomal instability, premature aging and cancer. We recently discovered novel mutations in the *RECQL4* gene in glioblastoma (GBM), the most malignant brain tumor in adults. Transcriptomic profiles of GBMs with REQCL4 mutations resembled those in REQCL4 KO glioma cells. We employ structural modelling and biochemical approaches to elucidate impacts of novel mutations on RECQL4 helicase activities. Using recombinant RECQL4_P532S_ and RECQL4_R766Q_ proteins we demonstrate that P532S substitution reduces the RECQL4 ability to unwind DNA and disrupts DNA-coupled ATP-hydrolysis activity. WT and mutated *RECQL4* were overexpressed in RECQL4 KO glioma cells to study interactions with BLM helicases, cell viability and specific responses to UVC- and chemotherapy-induced DNA damage/repair. Overexpression of RECQL4_P532S_ or RECQL4_R766Q_ variants affected DNA repair and responses to chemotherapeutics in glioma cells, and RECQL4_R766Q_ disturbed interactions with the BLM helicase. Our results reveal deleterious consequences of novel *RECQL4* mutations in GBMs. The newly identified *RECQL4* mutations affect RECQL4 helicases and their interactions with BLM contributing to glioma progression.

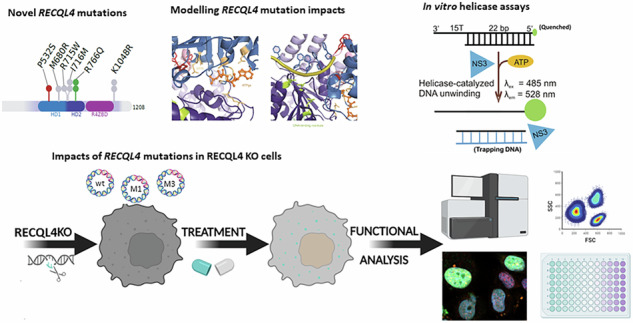

## Introduction

The RECQL4 helicase belongs to the highly conserved RecQ family of DNA helicases [1] that are involved in the ATP-dependent DNA unwinding and the maintenance of genome integrity. RecQ helicases preferentially bind to single-stranded DNA (ssDNA) translocating typically in a 3′ → 5′ direction. This binding and translocation are crucial for their function in various cellular processes such as genome replication, DNA repair, recombination, transcription, and translation. ssDNA and single-stranded DNA-binding proteins stimulate the unwinding activity of RecQ helicases on double-stranded DNA [2–4]. RECQL4 accumulates at foci formed by PML (pro-myelocytic leukemia) bodies and Rad51 at regions of single-stranded DNA upon induction of DNA damage to mediate homologous recombination [5]. RECQL4 knockdown in HeLa cells caused chromosome misalignment and delayed mitotic progression, independently from the RECQL4 role in DNA replication and damage repair [4]. Germline defects in *RECQL4* and other genes from the family: *RECQL1, BLM*, *WRN* results in syndromes associated with premature aging and predisposition to cancer [6, 7]. Mice with mutated or targeted deletions of *Recql4* rarely survive and those surviving show hallmarks of premature aging. Somatic deletion of *Recql4* in the adult mice results in severe hematopoietic deficiencies [8]*.*

Glioblastoma (GBM) is an aggressive, primary brain tumors, characterized by high proliferation, infiltrative growth, genomic instability, aberrant angiogenesis and resistance to major therapies [9]. Despite surgical resection, radio- and chemotherapy with temozolomide (TMZ), prognosis remains poor with a median survival of 14.6 months after diagnosis [10]. We found RECQL4 and BLM expression upregulated at mRNA and protein levels in GBMs and glioma cell lines. Depletion of RECQL4 or BLM in human glioma cells by CRISPR/Cas9 editing resulted in profound changes in the transcriptome, reduction of glioma sphere formation and augmented vulnerability to chemotherapeutics [11, 12]. Depletion of BLM helicase increased drug-induced polyploidization or cellular senescence depending on the TP53 status, and suggested the role of helicases in life-death decision making [12]. All these results emphasize the importance of RecQ helicases in malignant glioma progression.

We performed targeted next generation sequencing of 664 cancer-related genes in a large Polish Glioma Cohort and identified novel, potentially pathogenic variants in the *RECQL4* gene [13]. To comprehend the functional consequences of the recurrent *RECQL4* mutations in glioma pathogenesis, we combined genetic, biochemical, and cell biology approaches. The recently determined structure of human RECQL4_427-1116_ [14] allowed us to propose a mechanistic model in which the identified variants affect the evolutionarily conserved WD1 and WD2 helicase domains of RECQL4. We experimentally verified that the P532S substitution of a highly conserved proline impairs the ability of RECQL4 to unwind DNA by disrupting its DNA-coupled ATP-hydrolysis activity. The R766Q variant disturbed the regulatory network of RECQL4 and its interactions with BLM, affecting drug-induced cell death. Foremost, our work identifies two distinct molecular routes by which single point mutations in the *RECQL4* gene can contribute to GBM progression, providing additional insights for improvements in diagnostics and potential therapy.

## Results

### Characterization of novel *RECQL4* mutations identified in human glioblastomas

Using targeted next generation sequencing of a large cohort of gliomas (180 samples of different WHO grades) with a custom panel of 664 cancer- and epigenetics-related genes, we searched for genetic alterations and discovered a spectrum of pathogenic somatic and germline variants [13]. We identified a set of recurrent *RECQL4* mutations occurring only in glioblastoma which encompasses several novel, potentially pathogenic variants, for which no relevant information was found in several databases (e.g. National Center for Biotechnology Information, Ensembl, ClinVar). The *RECQL4* variants had minor allele frequency (MAF) < 0.001 in all examined databases, including ExAC and gnomAD databases. The reported *RECQL4* variants were substitutions, three of them were found in two patients. The presence of variants has been verified by targeted Sanger sequencing. An additional search found the variants in the dbSNP database (s763278718, rs931761657, rs772168426, rs202203322, rs373292946).

**Fig. 1:**
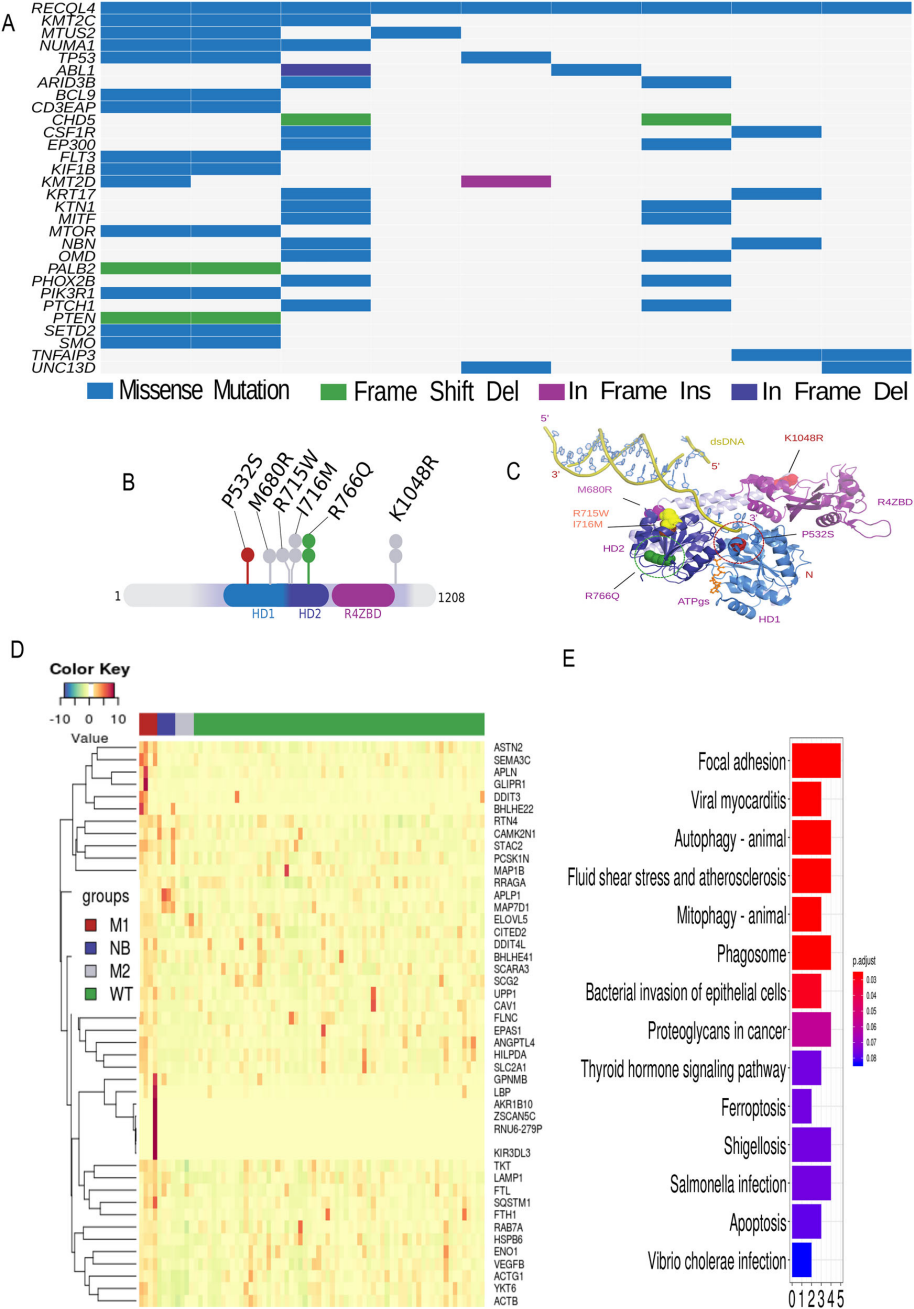
Identification of novel *RECQL4* mutations in glioblastomas. **A** The oncoplot summarizing the genetic alterations of top 30 genes associated with *RECQL4* mutations across all 9 GBM *RECQL4*-mutated samples. **B** RECQL4 lolliplot showing the identified substitutions mapped on the protein structure of the RECQL4. **C** Structure of human RECQL4_427-1116_ (pdb 5LST) superimposed with dsDNA from *H.sapiens* RECQL1 (pdb 2WWY) and ATPγs from *E.coli* RecQ (pdb, 1OYY) in a cartoon representation. Identified substitutions were mapped on the protein structure of RECQL4. A number of dots represents the number of patients in which the particular alteration was found. **D** Heatmap representing analysis of differentially expressed genes in 9 GBM samples with mutated RECQL4 (M1 defined as strong effect mutations and M2 as potentially silent mutations) versus normal brain (NB) and 64 GBM samples. **E** KEGG analysis of differentially genes (FDR corrected p < 0.1) showing upregulated expression in *RECQL4* mutated samples when compared to WT samples. X-axis scale shows a number of genes that were enriched in a given pathway. FDR-corrected p-value below 0.1 was considered significant.

The human *RECQL4* gene encodes a 1208 amino acid polypeptide, resulting in a 133 kDa protein with a highly conserved ATPase domain located at its center. The newly discovered mutations in the *RECQL4* gene lead to substitutions of single amino acids within the ATPase domain, comprising helicase domains (HD)1 and HD2, which are highly conserved among all RecQ-family proteins. Moreover, *RECQL4* mutations often co-occur with other mutated genes, however those samples were not classified as hypermutated GBM samples (Fig. 1A). The positions of substitutions in the RECQL4 protein sequence are shown using the Loliplot visualization (Fig. 1B). A potential impact of mutations on protein dynamics and stability was assessed with the web server DynaMut [15]. The identified mutations the *RECQL4* gene have potential to change its structural conformation and alter the associated functions (Fig. 1B, C). Four of the mutations were predicted to have a strong effect (RECQL4_P532S_, M1 set) while the effects of two substitutions (RECQL4_R766Q,_ M2 set) were undetermined (Fig. 1D).

Having RNAseq data for all tested samples, we next examined if the presence of mutations affects transcriptomic profiles in GBMs with the mutated *RECQL4* gene. A heatmap highlights that four M1 set samples show strikingly different patterns of expressed genes in comparison to the wild type (WT) RECQL4 glioblastoma samples or normal brains (NB) (Fig. 1D). A KEGG pathway analysis of differentially expressed genes in the M1 set GBMs showed the enrichment of genes related to focal adhesion, shear stress, proteoglycans in cancer and autophagy (Fig. 1E). Notably, these genes belong to similar gene categories as genes which were previously identified as significantly changed in RECQL4 knockout glioma cells [11] suggesting that the *RECQL4* variants could be loss-of-function mutations.

### Structural mapping of GBM-derived *RECQL4* mutations

**Fig. 2:**
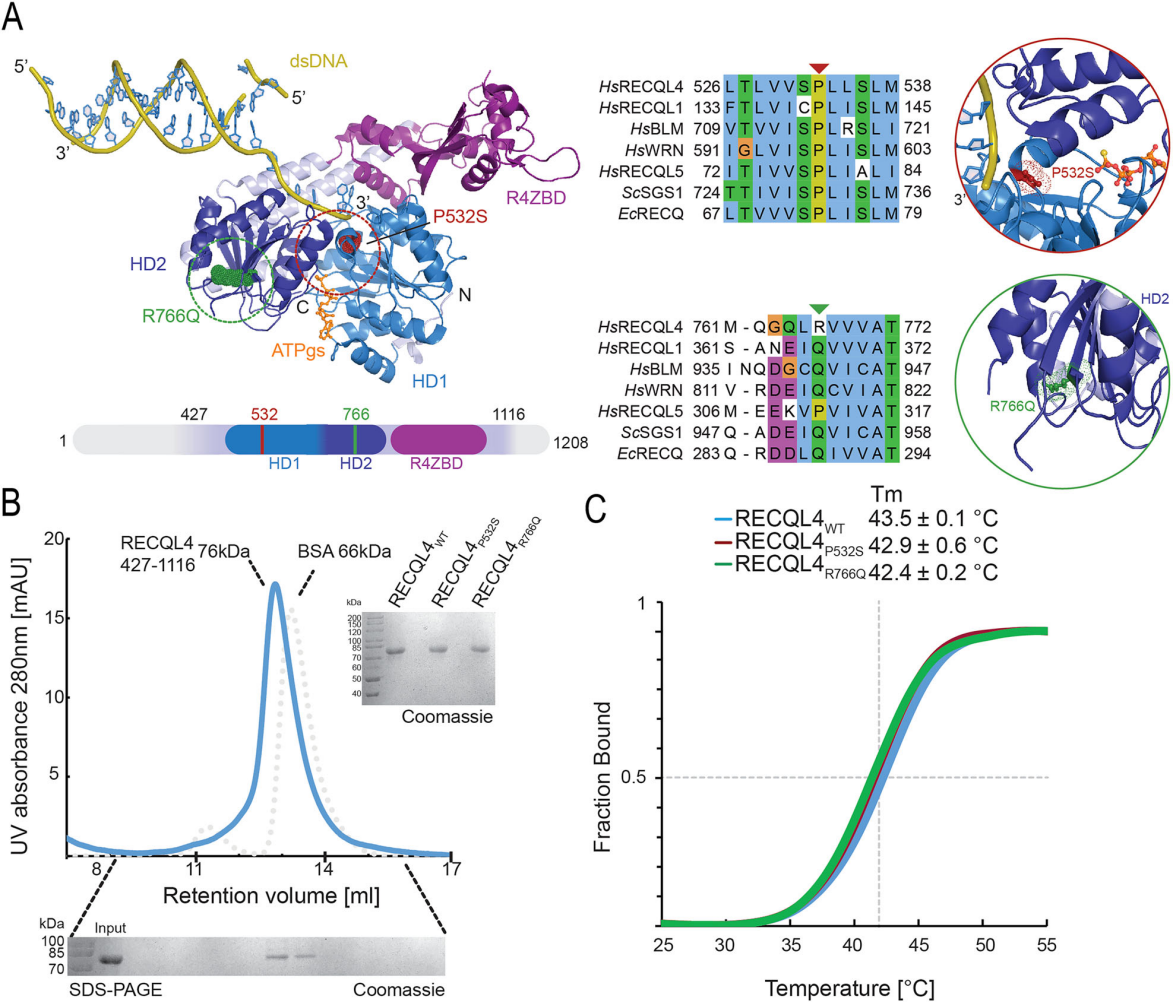
Modelling the structure of wild type RECQL4_427-1116_, P532S and R766Q recombinant proteins. **A** Structure of the human RECQL4_427-1116_ from Fig. 1. Positions of P532S and R766Q mutations are highlighted (left). Protein multiple sequence alignment of helicases, in areas proximal to the mutation sites. Human helicases RECQL1-5, as well as yeast SGS1 and bacterial RECQ are displayed (Uniprot ID: RECQL4-O94761, RECQL1-P46063, BLM-P54132, WRN-Q14191, RECQL5-O94762, SGS1-P35187, RECQ-P15043). Arrowheads highlight mutation sites (middle). Close-up view of the immediate area around the mutation sites. P532S is located in proximity to putative dsDNA and ATP binding sites. R766Q is located on a flexible, solvent-exposed loop (right). **B** Purification of the RECQL4_WT_ 427-1116 and RECQL4_P532S/R766Q_ proteins. High-purity protein samples were obtained after the final size-exclusion chromatography step, validated by SDS-PAGE and Coomassie staining. **C** Thermal shift assay on purified samples of RECQL4. All the proteins exhibit similar thermal stability. Experiments were performed on three independent protein isolations.

The available crystal structure of human RECQL4_427-1116_ [14] permits the precise localization of the P532S and R766Q variants in the enzymatic ATPase domain. As there is no available structure of the human RECQL4 bound to DNA, we used the evolutionarily conserved HD1 and HD2 domains [16] as well as ATP bound protein from *E.coli* RecQ [17] as the reference for the superposition (Fig. 2A). The quality of the superimposed models was validated by their similarity with independently obtained models from AlphaFold3 [18] and by analyzing various highly conserved residues derived from structures of RECQ-family proteins and associated with DNA binding or ATP-hydrolysis (Fig. 2B, C). The modelled ATP molecule is expected to accurately represent the native binding site of nucleoside triphosphates in RECQL4, due to its coordination by conserved residues within the well described P-loop motif (Supplementary Figure S1A). The position of the DNA in the RECQL4_427-1116_ structures carries a certain degree of uncertainty, largely owing to the inherent flexibility of the RECQL domains, and conformational changes upon DNA-binding [19]. RECQL4 lacks a well-defined β-hairpin motif (present in RECQL1, WRN, BLM) which is involved in strand separation of the double stranded (ds)DNA substrate. Nevertheless, many conserved residues, that are involved in single stranded DNA (ssDNA) binding, fall proximal to the modelled DNA (Supplementary Figure S1B). In particular, residues within the aromatic-rich loop (ARL) (Supplementary Fig. S1C**)** are proximal to the 3’ end of the ssDNA. Substitutions of residues within the ARL are known to abrogate the ability of RecQ proteins to unwind DNA [20, 21]. Interestingly, loss of ARL residues decreases DNA-dependent ATP-hydrolysis rates and at the same time increase basal ATP-hydrolysis rates when no DNA is bound [21]. Therefore, the ARL is considered a key motif responsible for coupling DNA-binding and hydrolysis of ATP to exert pulling forces on the ssDNA, which in turn drive the unwinding activity of the helicase.

The P532 residue, despite its localization between the P-loop motif and the ARL, has not yet been characterized functionally. The highly conserved residue E606, responsible for Mg^2+^ binding and ATP hydrolysis, is placed opposite P532. Readjustment of this residue together with the ARL are considered key for nucleic-acid dependent ATPase activity of helicases [21, 22]. Prolines are have rigid conformations needed to induce sharp turns in protein structures. Considering the necessity to couple an ATP-hydrolysis to the shift of ssDNA regulated by ARL, P532 could provide the conformational rigidity necessary for helicase activity. Unlike P532, R766 is not conserved within the RecQ family, and is placed distantly from the ATP-binding site or the DNA binding interface. Therefore, it seems less likely for R766 to be directly involved in the enzymatic RECQL4 activity. Nevertheless, its presence on a solvent exposed β-strand indicates its involvement in protein-protein interactions. Interestingly, the R766Q variant recapitulates a glutamine residue that is present and highly conserved in other members of the RecQ family. This indicates a novel gain-of-function variant of RECQL4_R766Q_, potentially inducing a change in specificity.

### Mutations in RECQL4 affect the helicase ATP hydrolyzing activity and binding to double stranded DNA

To examine properties of the RECQL4 P532S/R766Q variants, we employed the baculovirus expression vector system (BEVs) for recombinant protein production. Combining BEVS with the previously established purification protocol [14] we generated highly pure RECQL4 WT/P532S/R766Q proteins. The final size-exclusion chromatography profile was compared to BSA (bovine serum albumin) to confirm the monomeric nature of the sample and protein purity was assessed on Coomassie-stained SDS-PAGE gel (Fig. 2B). We performed a thermal stability shift assay (Fig. 2C) to check if the two *RECQL4* point-mutations do not affect overall folding and stability of the protein. Both RECQL4 variants exhibited very similar thermal stability parameters to the WT RECQL4, indicating that the identified substitutions do not result in protein instability or proteolysis.

**Fig. 3:**
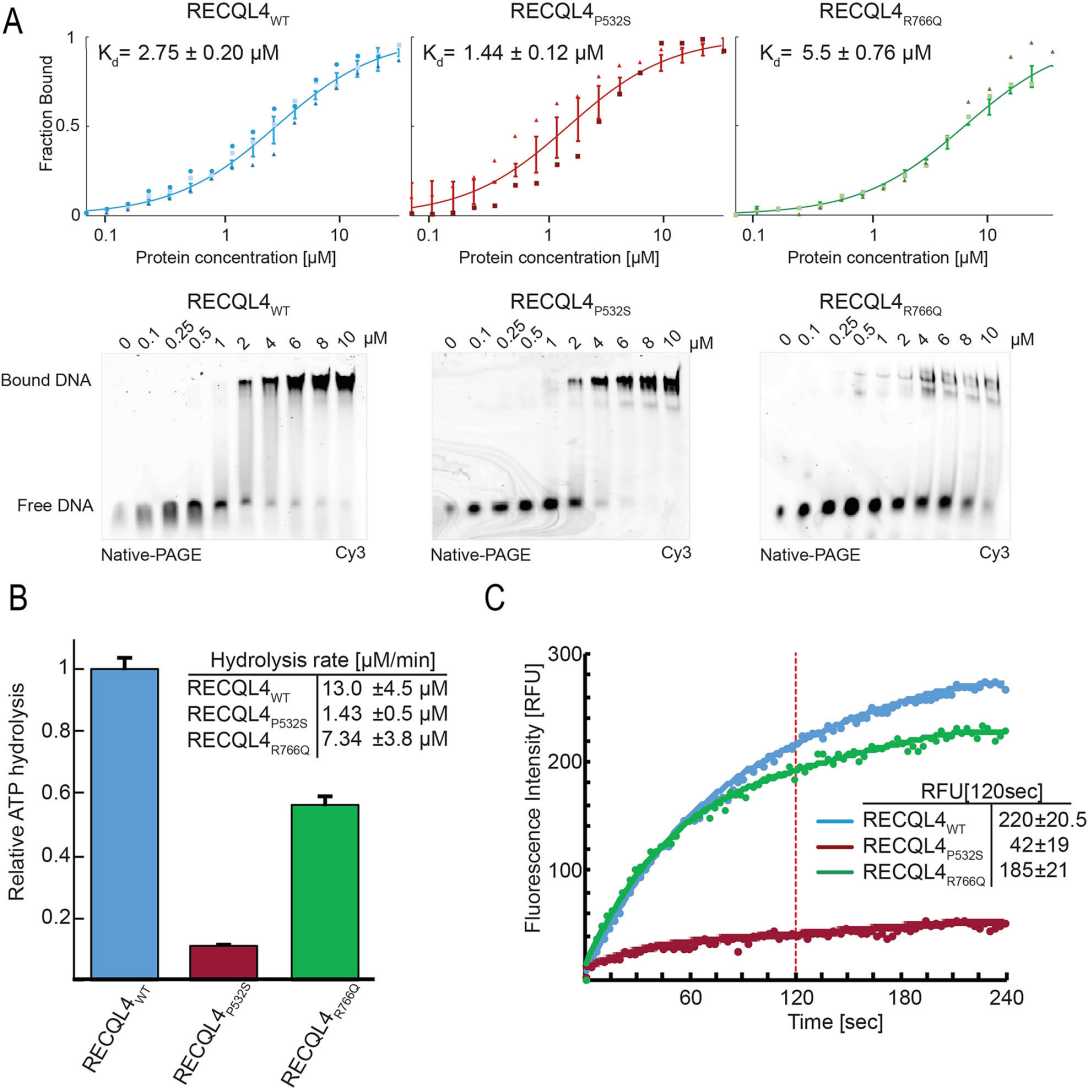
Biochemical characterization of RECQL4 mutants. **A** DNA binding affinity of RECQL4 to the dsDNA measured via MST (top) and EMSA (bottom). K_d_ values were calculated from the MST data (n = 3). **B** ATP hydrolysis of RECQL4 in the presence of dsDNA, quantified by a malachite green assay. Results were normalized to the hydrolysis rate of RECQL4_WT_ after subtraction of basal ATP hydrolysis with no protein present. Calculated ATP hydrolysis rates for 1 μM of RECQL4 are shown as mean ± SD, n = 3, in duplicates. **C** Enzymatic activity of RECQL4, measured with the helicase assay. After addition of ATP, helicase separates Cy3 labelled strand from the complementary quencher, resulting in an increase in fluorescence. Results were quantified by comparing the detected fluorescent intensity 2 min after ATP addition and are shown as mean ± SD, n = 3, in triplicates.

Two independent DNA binding affinity assays were performed to characterize the impact of the P532S/R766Q substitutions on RECQL4 helicase functions (Fig. 3A). An EMSA assay was employed to test complex formation on DNA and a quantitative microscale thermophoresis (MST) assay to determine specific binding parameters. We used the 15 nucleotide-long double-stranded oligo with a single strand 15 nucleotide polyA extension as a model DNA-substrate [14]. We found significant differences in DNA affinity for both mutants compared to WT RECQL4, with P532S exhibiting a stronger affinity and R766Q displaying a weaker affinity. These results highlight the strong consequences of mutations.

Since the separation of a single base-pairing bond in dsDNA requires additional energy from NTP hydrolysis, we performed a malachite green-based ATPase assay, examining the ability of RECQL4 to hydrolyze ATP in the presence of dsDNA binding (Fig. 3B). Strikingly, the P532S mutation caused a sharp decrease in the ATP hydrolysis rate, in comparison to RECQL4_WT_ and RECQL4_R766Q_ proteins. Notably, the RECQL4_P532S_ mutation causes a significant loss in the capacity of RECQL4 to hydrolyze ATP, despite its increased affinity towards the DNA substrate.

Finally, we tested the enzymatic activity of RECQL4 using an in vitro helicase assay [14] (Fig. 3C). In this fluorescence-based assay, the individual strands of dsDNA are separated by the RECQL4 helicase, liberating the fluorescent tag from its complementary quencher, resulting in increased fluorescence. The RECQL4 P532S was unable to unwind the dsDNA substrate. These results extend the network of critical conserved residues in RECQ-like helicases and tie them directly to an observed phenotype. The stronger affinity of RECQL4_P532S_ to dsDNA is surprising, as previous results indicated a direct correlation between an increase in the helicase rates of RECQL4 and its affinity for DNA [14]. Taken together, we demonstrate that the novel single-point mutations influence the enzymatic activity of RECQL4 in vitro. In particular, the P532S mutation has drastic effects on the RECQL4 helicase activity.

### RECQL4 variants differently interact with the BLM helicase in human glioma cells

**Fig. 4:**
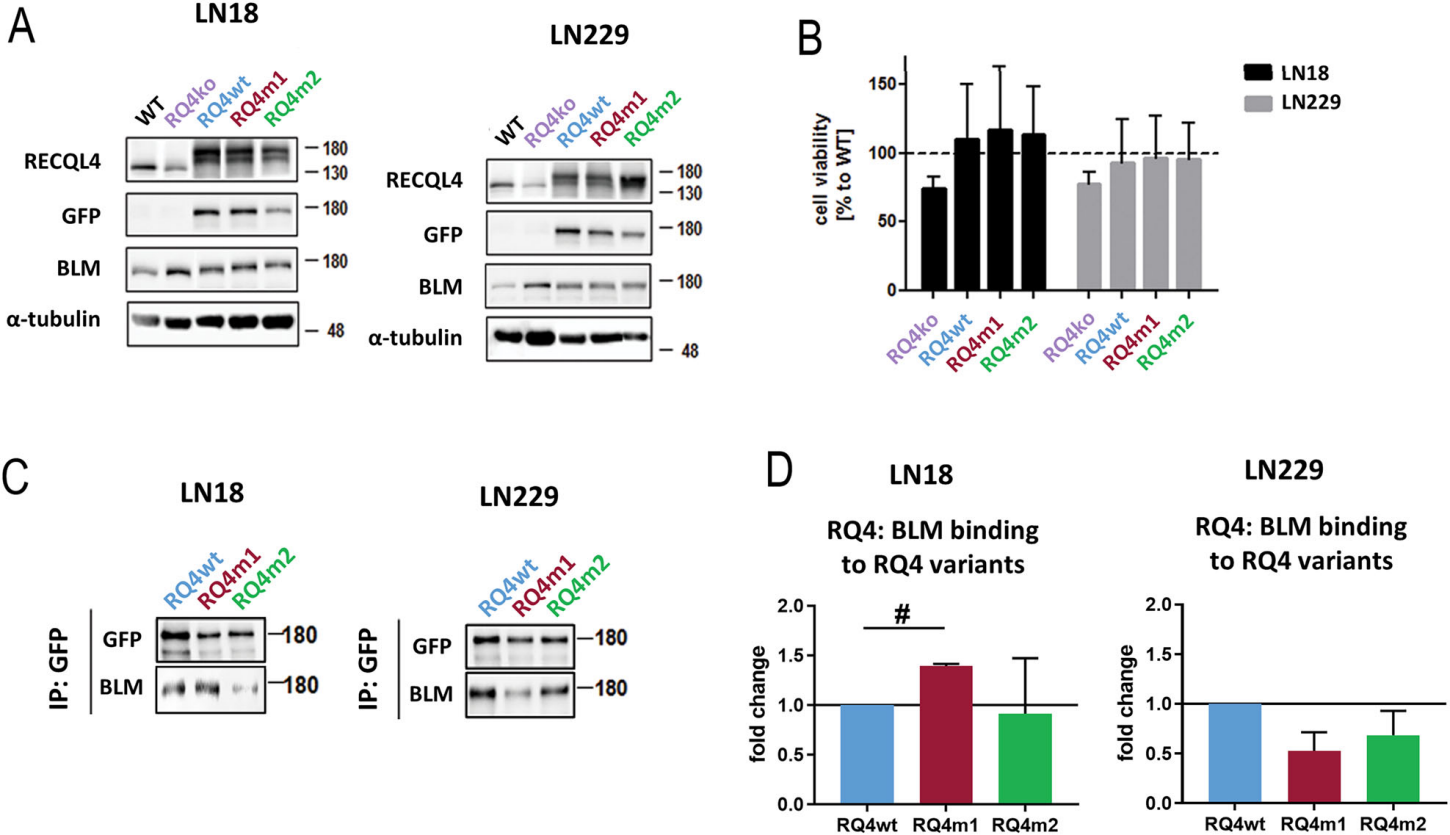
RECQL4 variants compensate for RECQL4 deficiency in cell viability assay but differently interact with BLM helicase. **A** Representative immunoblots show GFP-tagged WT RECQL4 (RQ4wt), RECQL4_P532S_ (RQ4m1) and RECQL4_R766Q_ (RQ4m2) proteins overexpressed in human RQ4 KO LN18 and LN229 glioma cells detected by Western blotting. The presence of GFP resulted in a high molecular weight of the RECQL4 proteins. The levels of BLM helicase and a-tubulin are shown. **B** Cell viability was determined with PRESTO Blue 48 h after transfection of RECQL4 KO cells with the tested RECQL4 constructs. The results are shown as mean ± SD, n = 4, in triplicates. **C**, **D** Immunoprecipitation experiment showing distinct binding RQ4 variants to its partner BLM in LN18 and LN229 glioma cells (**C**) and its quantification (**D**). RECQL4 deficient cells were transfected with the tested RECQL4 constructs and 48 h after transfection cells were lysed, total protein extracts isolated and subjected to immunoprecipitation (IP) with anti-GFP antibody. IP extracts were resolved by SDS-PAGE and subjected to Western blotting. Immunoblots were quantified by densitometry, results are presented as mean ± SD, n = 3. Statistical analysis was performed on log raw data using one-way ANOVA with Tukey’s post-hoc test (# p < 0.001). Uncropped Western blots are presented in the Supplemental material.

To study RECQL4 functions in human cells, we employed previously generated human LN18 and LN229 glioma cells depleted of RECQL4 (RQ4 KO) [11]. RECQL4 variants were generated by site directed mutagenesis. The constructs coding for the WT RECQL4 (RQ4wt), RECQL4_P532S_ (RQ4m1) and RECQL4_R766Q_ (RQ4m2) linked to GFP were overexpressed in RECQL4 depleted LN18 and LN229 glioma cells. The levels of RECQL4 and GFP in transfected cells were quantified by Western blotting (Fig. 4A). The transfection efficiency was comparable in case of all RECQ4 variants. RECQL4 KO cells show upregulation of the BLM helicase and overexpression of any of all three constructs blocks this upregulation and restores basal BLM levels. Depletion of RECQL4 in LN18 and LN229 glioma cells reduced cell viability by 20% and overexpression of all tested RECQL4 constructs improved cell viability (Fig. 4B). RECQL4_P532S_ (RQ4m1) and RECQL4_R766Q_ (RQ4m2) behave like RQ4wt and this lack of difference shows that they compensate similarly when cell viability is considered.

To determine how RECQL4 variants interact with other helicases [23], we overexpressed RQ4wt, RQm1 and RQ4m2 in RQ4 KO LN18 and LN229 cells, and after 24 h the complexes of RECQL4-GFP with interacting proteins were immunoprecipitated (IP) with an antibody against GFP. The cells transfected with GFP alone did not show a non-specific signal with the BLM or RECQL4 antibodies (Supplementary figure S2, Supplemental material) The presence of BLM in the IP complexes was detected by Western blotting. The interaction of RQ4m1 with the BLM helicase was impaired in LN18 glioma cells (Fig. 4C, D, Supplemental material), while in LN229 cells this binding was decreased, although insignificantly (Fig. 4C, D). BLM was equally bound by the RQ4m2 variant in RQ4 KO LN18 and LN229 cells. The results were corroborated by densitometric analysis of immunoblots from 3 independent experiments (Fig. 4C, D).

**Fig. 5:**
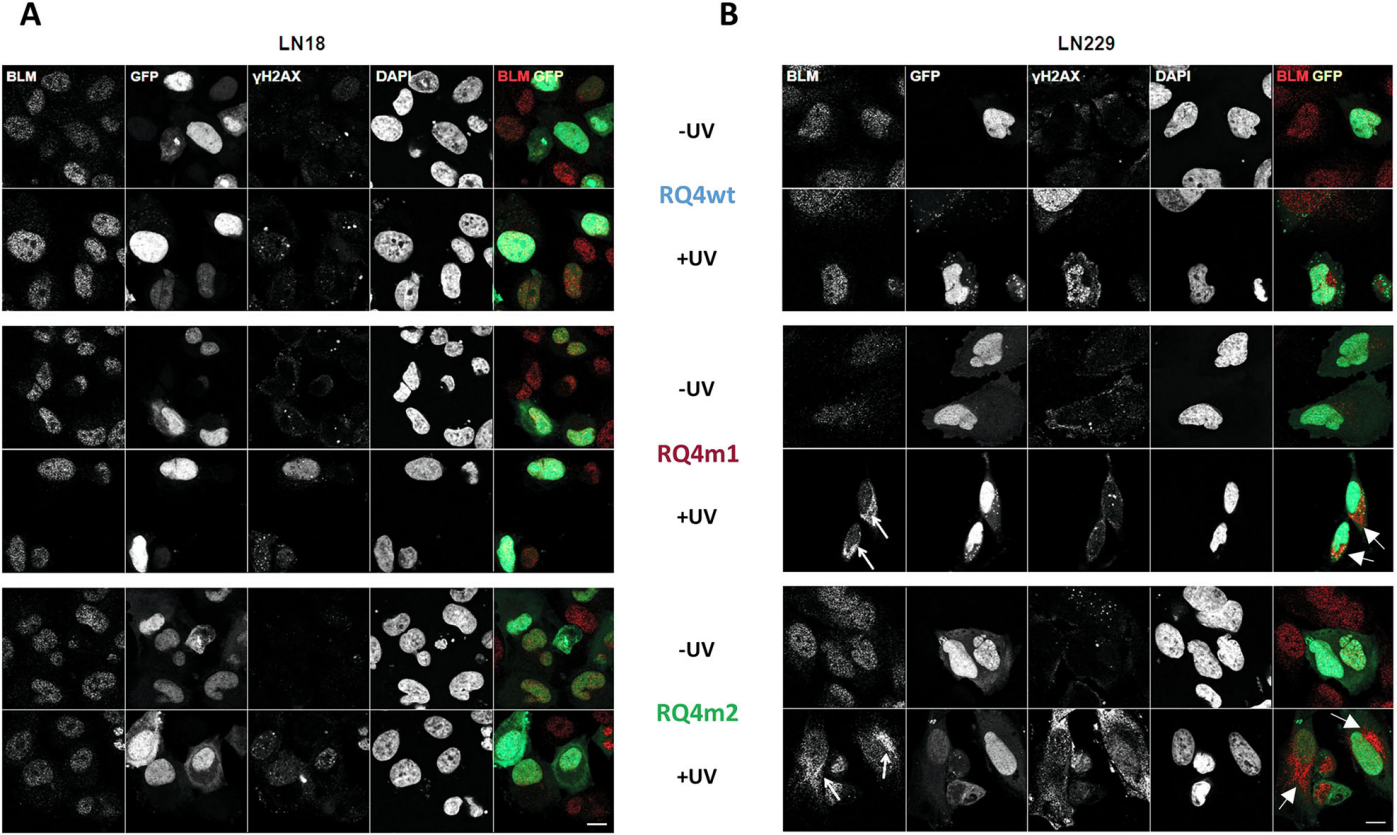
Subcellular localization of GFP-RECQL4 variants under basal conditions and after UV-C irradiation. **A, B** Immunofluorescent staining showing localization of BLM, different GFP-RECQL4 variants and γH2AX in RECQL4 KO LN18 **(A)** and LN229 **(B)** glioma cells under basal conditions and after exposure to UV-C. Nuclei were visualized by DAPI staining and γH2AX foci accumulate at the sites of double stand DNA breaks. White arrow indicate BLM sequestered in the cytoplasm in UV-C treated cells transfected with GFP-tagged RECQL4_P532S_ (RQ4m1) and RECQL4_R766Q_ (RQ4m2) proteins. Scale bar 50 µM. Images are representative of 3 independent experiments.

RECQL4 and BLM helicases are involved in repair of DNA double-strand breaks by the homologous recombination (HR) pathway. During HR, RECQL helicases have both pro- and anti-recombinogenic activities, which may contribute to the maintenance of genomic integrity [23]. To study if *RECQL4* mutations affect those processes, the constructs carrying WT or mutated *RECQL4-GFP* genes were overexpressed in RQ4 KO glioma cells and the cells were exposed to UV-C light which introduces double strand breaks (DSBs) into DNA. DSBs were visualized by staining of γH2AX, a protein which accumulates at DSB sites [24]. We have previously shown that BLM co-localizes with γH2AX in UV-C irradiated human glioma cells [12]. Overexpression of RECQL4-GFP variants in LN18 cells did not change the appearance of γH2AX and BLM foci in UVC-treated glioma cells (Fig. 5A). However, overexpression of both RQ4m1 and RQ4m2 variants led to sequesteration of the BLM protein from the nuclei to the cytoplasm in UV-C treated cells (indicated by white arrows). The RQ4wt did not change the nuclear localization of BLM (Fig. 5B). These results show that RECQL4 variants could restrict BLM helicases in the cytoplasm of human glioma cells undergoing DNA damage preventing their action in DNA repair and translocation to DSB sites, indicated by the γH2AX foci.

### Overexpression of RECQL4 variants reduces chemotherapeutics-induced cell death of human glioma cells

Most of anti-tumor therapies eliminate neoplastic cells by introducing DNA damage which ultimately triggers cell death. These effects are counteracted by activated DNA repair pathways that efficiently diminish therapeutic effects [25, 26]. We determined if RECQL4 variants (which altered interactions with BLM and DNA repair) affect glioma cell responses to TMZ, a main chemotherapeutic in glioma therapy. We performed immunofluorescent staining of γH2AX and BLM in RECQL4 KO cells expressing RECQL4 variants after treatments with DMSO (a solvent) or TMZ. Immunofluorescence signal of BLM and γH2AX in cells exposed to various conditions was quantified.

**Fig. 6:**
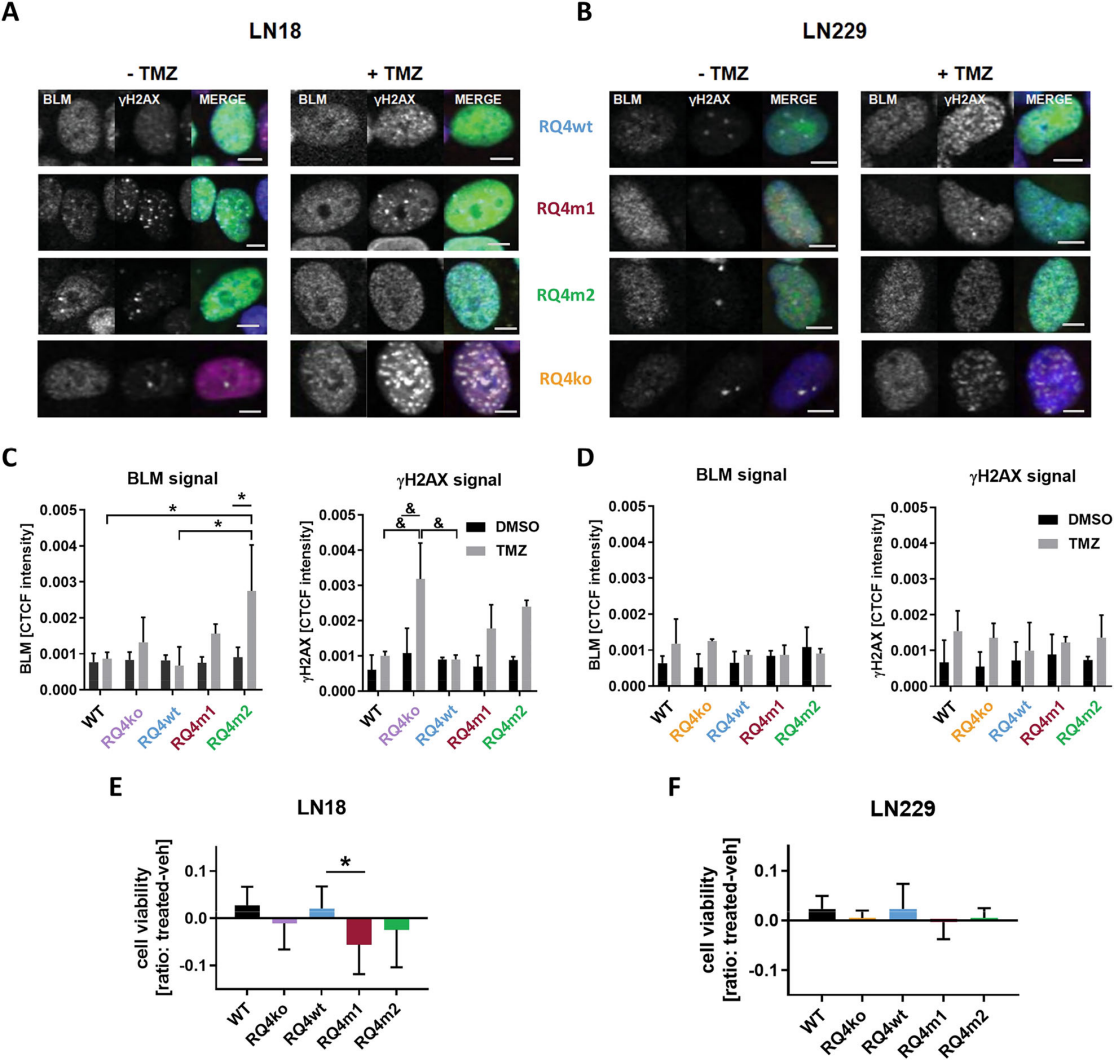
The impact of GFP-RECQL4 variants on BLM and γH2AX signal intensity in TMZ-treated glioma cells. **A**, **B** Immunofluorescent staining showing expression of BLM, and γH2AX in RECQL4 KO LN18 **(A)** and LN229 (**B**) glioma cells overexpressing GFP-RECQL4 variants under basal conditions and after exposure to 500 µM TMZ for 48 h. TMZ induces γH2AX accumulation in KO cells transfected with GFP-tagged wild type RECQL4 (RQ4wt) but not in cells expressing RECQL4_P532S_ (RQ4m1) and RECQL4_R766Q_ (RQ4m2) proteins. TMZ induces a prominent γH2AX accumulation in RECQL4 KO LN18 cells and moderate γH2AX accumulation in RECQL4 KO LN229 cells. Scale bar 50 µM. Images are representative of 3 independent experiments. **C**, **D** Quantification of BLM and γH2AX in RECQL4 KO LN18 (**C**) and LN229 **(D**) glioma cells overexpressing GFP-RECQL4 variants. Immunofluorescence signal intensity was measured. **C** BLM signal intensity is significantly increased in TMZ-treated RECQL4 KO LN18 cells expressing RECQL4_R766Q_ (RQ4m2) proteins. TMZ-induced γH2AX accumulation in LN18 RECQL4 KO cells is counteracted by transfection with RQ4wt but not with RECQL4_P532S_ (RQ4m1) and RECQL4_R766Q_ (RQ4m2). **D** TMZ does not induces similar changes in LN229 that show γH2AX accumulation after TMZ treatment. Results are presented as mean ± SD, n = 3. WT –parental cells. Statistical analysis was performed on raw data using two-way ANOVA with Tukey’s post-hoc test (within the groups) or Sidak’s post-hoc test (between DMSO and TMZ groups) (*p < 0.05, & p < 0.01). **E**, **F** Cell viability of RECQL4 KO LN18 (**E**) and LN229 (**F**) glioma cells overexpressing GFP-RECQL4 variants and treated with TMZ. Cell viability was determined with Presto Blue. Results are presented as mean ± SD, n = 3. WT –parental cells. Statistical analysis was performed on raw data using one-way ANOVA with Dunnett’s post-hoc test (*p < 0.05).

The γH2AX foci appeared in the nuclei of RQ4 KO LN18 glioma cells exposed to TMZ indicating sites of drug-induced DNA damage (Fig. 6A, C). Overexpression of the RQ4wt reduced this effect showing its capacity to contribute to DNA repair. The Fig. 6C illustrates that in TMZ treated LN18 cultures, where an intensive DNA damage/repair takes place, overexpression of RQ4m2 leads to the augmented presence of BLM at the DNA damage foci (manifested by the higher intensity of the BLM signal). This is a consequence of increased binding of BLM by the mutant and may contribute to enhanced repair of DNA alleviating the TMZ induced damage. Next panel shows accumulation of γH2AX in TMZ treated KO cells, its reduction in cells overexpressing RQ4wt but not RQ4 mutants which suggests that due to increased binding of BLM, RQ4 mutants interfere with the formation of γH2AX foci. In LN229 cells, where most cells die by apoptosis after TMZ, overexpression of RECQL4 variants produced weaker effects on the BLM signal and γH2AX foci in TMZ-treated glioma cells, and the changes were not significant (Fig. 6B,D). Overexpression of the RQ4wt reduced this effect showing its capacity to contribute to DNA repair. RQ4m1 and RQ4m2 variants were less effective. Overexpression of RQ4wt in RECQL4 KO LN18 cells restored their resistance to TMZ as evidenced by the improved cell viability, suggesting that RQ4m1 and RQ4m2 variants did not protect RECQL4 KO cells against TMZ (Fig. 6E). Similar trends (but not significant) were observed in LN229 cells (Fig. 6F) which are sensitive to TMZ irrespectively of RECQL4 levels. These results demonstrate that RECQL4 variants are unable to compensate for RECQL4 loss during drug-induced DNA damage, restrict BLM accessibility in the nucleus which may result in impairments of DNA repair processes after TMZ making cells more vulnerable the drug-induced DNA damage.

## Discussion

The RECQL4 helicase is responsible for preventing multiple clinical syndromes, cancer and premature aging but its role has not been clarified. The detection of clinically relevant *RECQL4* mutations demonstrated the dual roles of RECQL4 helicases: first in maintaining the genome integrity, secondly in DNA repair which determines responses to therapeutic drugs in the case of cancer. RECQL4 is highly overexpressed in many cancers but it is unknown whether RECQL4 acts as a tumor suppressor or an oncogene. Defining the molecular consequences of different *RECQL4* mutations is imperative to expand our understanding of clinical phenotypes, and the dual roles of RECQ4 in cancer development and suppression. Glioblastomas are deadly and currently incurable tumors, thus identifying genetic alterations underlying tumorigenesis and having an impact on responses of GBM cells to therapy is of clinical importance.

Here, we report the discovery of novel, recurrent mutations in the *RECQL4* gene in GBM patients. We focused on the consequences of two, recurrent single-point mutations on the enzymatic RECQL4 activity and its functions in human glioma cells. We show that the P532S alteration affects the RECQL4 helicase activity. It is interesting that the RECQKL4_P532S_ affinity for DNA is similar to the RECQL4_WT_, which means that tightly DNA-bound RECQL4_P532S_ might create road blocks in the genome that are cannot be resolved or released after the initial binding step. The highly evolutionarily conserved domains HD1 and HD2 permitted the analysis of P532S and R766Q, and their effects on the enzymatic core of RECQL4. Within the Sf1 helicase family, PcrA and UvrD motifs are important for the ATP-binding induced rotation of the HD domains and closure of the cleft between them, thereby allowing the enzyme to translocate DNA [27]. The obtained models of RECQL4 variants along with biochemical experiments on recombinant proteins clearly demonstrated a defective helicase activity and inability to unwind DNA along with differences in binding to DNA which may strongly affect cellular processes. Intriguingly, the distinctive spectrum of malignancies is associated with each of the RecQ deficiencies [28]. In Bloom’s syndrome, about half of all malignancies are epithelial carcinomas and the other half are lymphomas and leukemias. In Werner’s syndrome, half are thyroid cancers and melanomas representing epithelial tumors, whereas soft-tissue sarcomas and osteosarcomas represent the majority of non-epithelial tumors. In Rothmund-Thomson syndrome, skin cancers account for most of the tumors of epithelial origin while osteosarcomas represent the majority of the non-epithelial tumors [3, 29]. We show the presence of various mutations in the *RECQL4* in malignant gliomas of mesenchymal origin.

Biological consequences of *RECQL4* mutations in human glioma cells were evaluated in RECQL4 depleted glioma LN18 and LN229 cells generated by the CRISPR/Cas9 gene editing [11]. RECQL4 KO cells have cell viability reduced by 20%. Despite significant effects of the RQ4m1 (P532S substitution) on the RECQL4 activity in vitro, RECQL4_P532S_ behaves as RECQL4_WT_ in cell viability assay, restoring cell viability. However, we found that RECQL4_P532S_ binds strongly BLM in LN18 cells, and RECQL4_R766Q_ restricts BLM in the cytoplasm of LN229 glioma cells, particularly when DNA damage was inflicted by UV-C irradiation. Strong binding and sequestration of the BLM helicase in the cytoplasm may affect DNA repair processes and genome integrity contributing to tumorigenesis but on the other hand it makes cells more vulnerable to the treatment by reducing their abilities to DNA repair.

LN18 cells are insensitive to TMZ due to the lack of a functional TP53 and active MGMT repair system, however the RECQL4 deficiency makes them more sensitive, which is visualized by the increased signal intensity of γH2AX at the double stranded DNA breaks. Restoration of a wild type RECQL4 in those cells reestablishes resistance to TMZ. Overexpression of RECQL4 variants does not have such effects showing their failure in the proper DNA repair. We admit that some of the effects are rather weak (do not reach statistical significance) but this could be attributed to the fact that biological assays were carried out on transfected cells and cell transfectability of human glioma cells is moderate. We and others have shown that RECQL1 and RECQL4 helicases are overexpressed in gliomas which confers poor prognosis due to their abilities to support proliferation and DNA repair [11, 28]. Since the identification of the *RECQ4* gene in 1998, multiple *RECQ4* mutations linked to developmental alterations and the pathogenesis of Rothmund-Thomson syndrome, Baller-Gerold syndrome, and RAPADILINO syndromes have been identified. Moreover, a subset of *RECQ4* mutations is associated with high cancer risks [29], however, those clinically relevant *RECQ4* mutations in tumor cells are poorly studied.

We found that the RECQL4 variant shows the reduced helicase ATPase activity and differently interaction with DNA or exhibit different interactions with the BLM helicase restricting both proteins in the cytoplasm, those defects would result in reduced helicase activities in DNA repair processes. The defects might have deleterious effects on DNA stability and repair predisposing to cancer. On the other hand, the inability of RECQL4 variants to cope with DNA repair after drug-induced DNA damage may sensitize those cells to TMZ improving therapy outcomes. Clarification of the structure and functions of RECQL4, and discovering novel mutations may support the rationale for personalization of tumor therapy based on the genetic testing of the *RECQL4* gene.

In conclusion, we demonstrated that the deleterious consequences of newly discovered *RECQL4* mutations on an enzymatic activity and DNA damage/repair processes in cells. A substitution of a highly conserved proline in the RECQL4 P532S (RQ4m1) impairs the ability of RECQL4 to unwind DNA by disrupting the DNA-coupled ATP-hydrolysis. The R766Q (RQ4m2) variant did not affect the enzymatic activity of RECQL4 in vitro but disturbed the RECQL4 network and its interactions with BLM which results in cellular dysfunctions and altered vulnerability to chemotherapy.

## Materials and Methods

### Identification of somatic and rare germline variants in glioma samples

The analysis of *RECQL4* gene in a cohort encompassing 180 gliomas of different WHO grades was performed as previously described [13]. As a result of the computational pipeline only putative somatic and rare germline variants (less than 1 in 1000 cases in general population) were reported. The presence of *RECQL4* mutations was verified by Sanger sequencing (Genomed, Warsaw).

### Transcriptomic analysis

RNAseq data were analyzed as previously described [13] using the same cohort of samples with an additional 4 GBM samples with mutated *RECQL4*. Differential expression analysis was performed between RECQL4 WT, M1 or M2 groups. FDR-corrected p-values of 0.1 were considered as significant. Transcriptomic data were analyzed as follows: fastq files were aligned to hg38 human reference genome with STAR program [30], and reads were counted to genes using the feature Counts algorithm SUBREAD package [31]. Gene counts were normalized with the FPKM method, and differential analysis was performed using the NOIseq [32, 33]. Kyoto Encyclopedia of Genes and Genomes (KEGG) pathway analyses were performed using the R package clusterProfiler [34]) to annotate the functions of differentially expressed mRNAs.

### Expression and purification of human RECQL4 proteins

Codon-optimized human *RECQL4* gene (Genscript) was cloned as the RECQL4_427-1116_ truncated construct into pFastBacHTC vector for expression in the Bac-to-Bac Baculovirus Expression Vector System (BEVS), according to standard protocols. Mutations resulting in P532S and R766Q were introduced using primer-based mutagenesis. Hi5 insect cells were infected with a MOI = 0.5 and grown for 3 days at 27 °C. Cells were collected by centrifugation at 8000 × g for 10 min and resuspended in ice cold buffer A (30 mM HEPES pH 7.5, 300 mM NaCl, 5 mM MgCl_2_, 2 mM β-mercaptoethanol, 5% glycerol and protease inhibitors). After addition of DNAase and lysozyme, cells were lysed by three freeze-thaw cycles using liquid nitrogen and subsequently sonicated and centrifuged for 20 min at 20,000 × g. The supernatant was loaded on a pre-equilibrated GSTtrap^TM^ 5 mL (GE Healthcare) column on an ÄKTA start chromatography system (GE Healthcare), washed and eluted with 10 mM reduced glutathione. To remove bound nucleic acids and other contaminants, the pooled elution fractions were diluted in buffer B (30 mM HEPES pH 7.5, 30 mM NaCl, 5 mM MgCl_2_, 2 mM β-mercaptoethanol, 5% glycerol) to decrease the salt concentration ( < 100 mM), loaded on HiTrap^TM^ Heparin HP 5 ml column and eluted with a salt gradient. GST-tagged TEV protease was added to the eluates (ratio 1:100), loaded into dialysis cassettes (Slide-A-Lyzer; 10,000 MWCO) and incubated overnight (o/n) in dialysis buffer (30 mM HEPES pH 7.5, 300 mM NaCl, 5 mM MgCl_2_, 2 mM β-mercaptoethanol, 5% glycerol). Samples were removed from the dialysis cassettes and loaded again on pre-equilibrated GSTtrap^TM^ column to separate the cleaved RECQL4 proteins from GST tag and GST-TEV. The flowthrough was collected and concentrated by centrifugation using a concentrator (Amicon-Ultra, 30,000 MWCO (MERCK)), and loaded on a Superdex^TM^ 200 increase 10/300 GL (GE Healthcare) size-exclusion chromatography column pre-equilibrated with buffer C (10 mM HEPES pH 7.5, 100 mM NaCl, 5 mM MgCl_2_, 2 mM DTT, 5% glycerol). Protein purity and quality were validated by SDS-PAGE stained with Coomassie blue. Fractions containing pure RECQL4 protein were pooled, concentrated to >3 g/L, snap-frozen in liquid N_2_ and stored at -80 °C.

### Thermal shift assay

The thermostability of RECQL4_WT_ as well as RECQL4_P532S_ and RECQL4_R766Q_ mutants was evaluated using thermal shift assays. 20 μg of each protein sample were incubated in buffer C together with SYPRO Orange (Sigma Aldrich) hydrophobic fluorescent dye for 10 min. Samples were gradually heated up from 4-98 °C at a speed of 0.6 °C/min in a thermal cycler (Biorad). The fluorescent signal emitted by the probe was detected constantly throughout the whole thermal gradient.

### dsDNA substrate preparation and electrophoretic mobility shift assay

DNA oligonucleotides (biomers.net) were re-suspended in milliQ purified water to a concentration of 1 mM and stored at -20 °C. Oligonucleotide sequences were derived from (14): OH-Cy3/5, CCATTCCACCCTCTATTTTTTTTTTTTTTT, labelled with 5’ Cy3/Cy5 and CQ-Dab, TAGAGGGTGGAATGG, labelled with 3’ Dabcyl. Annealing of dsDNA was performed in buffer D (10 mM HEPES pH 7.5, 50 mM NaCl, 5 mM MgCl_2_, 2 mM DTT) before each experiment. 10 μM of complementary DNA strands were heated in 20 μL to 95 °C for 5 min and gradually cooled to 20 °C at a rate of 1 °C/min by a PCR thermal cycler (Eppendorf, Germany). Generated, annealed dsDNAs with single-stranded overhangs were stored at 4 °C in the dark until use.

To assess the DNA binding capacity, increasing concentrations of purified RECQL4 protein (0.1-10 μM) were incubated with 100 nM of Cy3-labelled dsDNA in buffer D. The bound and unbound fractions were separated by electrophoretic mobility shift assay using native-PAGE (6%). Cy3-labelled dsDNA was visualized with a fluorescent scan (Typhoon) at a wavelength of 580 nm.

### Microscale thermophoresis (MST), ATPase activity and helicase activity

The RECQL4-dsDNA binding was quantified via MST using standard methods. In brief, the protein gradient was incubated with 10 nM Cy5-labelled dsDNA and transferred in capillaries to Monolith NT.115 (Nanotemper). Individual MST runs are displayed with a fitted dose-response curve. The assay was performed in independent triplicates using at least two batches of purified protein.

ATP hydrolysis activities of RECQL4 proteins were assessed using Malachite Green Phosphate Assay kit (Sigma-Aldrich) according to manufacturer’s instructions. In detail, RECQL4 proteins (0.5 μM) were incubated for 15 min in buffer D supplemented with 1 mM ATP and 100 nM dsDNA at RT. Reactions were diluted in water and concentrations of free phosphate were measured via absorption at a wavelength of 620 nm and quantified with the use of a standard calibration curve.

To evaluate the capacity of RECQL4 to unwind dsDNA, helicase assays were performed as described (14). Complementary DNA strands were each conjugated to either a fluorescent tag (Cy3) or a respective quencher (Dabcyl). Unwinding of the pre-annealed dsDNA separates the Cy3 probe from its proximal quencher, resulting in an increase of fluorescence. 1 μM RECQL4 in buffer D was pre-incubated for 10 min with 50 nM Cy3/Dab-dsDNA. After addition of ATP to a final concentration of 1.25 mM, the fluorescence intensity was continuously measured for 4 min. Measurements without ATP addition served as a control and were subtracted from the observed signal. Results are shown as mean change in fluorescence intensity (FI), with a least-squares fitted curve displayed. Changes in FI after 2 min of incubation were used to quantify the differences between the RECQL4 protein variants. Data represent an average of at least three independent experiments in duplicates.

### Cell cultures and treatments

LN18 and LN229 glioma cell lines were purchased from the American Type Culture Collection (ATCC, Manassas, VA, USA) and cultured in DMEM medium (Dulbecco’s modified Eagle medium, Thermo Fisher Scientific; Waltham, MA, USA). LN18 and LN229 RECQL4 knockout cells (LN18 RQ4 KO, LN229 RQ4 KO) were previously generated using CRISPR/Cas9 genome editing and characterized as described [11]. Cells were maintained in DMEM culture media supplemented with 10% FBS (Gibco, USA), antibiotics (100 U/mL penicillin, 100 µg/mL streptomycin) in a humidified atmosphere of CO_2_/air (5%/95%) at 37 °C. Cells were treated with 500 µM temozolomide (TMZ, Sigma-Aldrich, Munich, Germany) dissolved in DMSO.

### Cloning and mutagenesis of RECQL4 variants

The human *RECQL4* gene (NCBI nucleotide accession number NM_004260.3) was cloned into the pcDNA3.1( + )-N-eGFP expression vector (Clone ID OHu21029D), which was purchased from Genscript. The *RECQL4* mutants were generated by mutagenesis of the wild type *RECQL4*. Briefly, the GFP tag was removed from the original N-GFP expressing plasmid using a PCR reaction with following primers: (Fwd: 5’-ATGGAGCGGCTGCGGGACG-3’, Rev: 5’-GGTGGCAAGCTTAAGTTTAAAC-3’). Next, the GFP fragment was amplified using the following primers (Fwd: 5’-ATCGAATTCAGCAAGGGCGAGGAGCTGTTCAC-3’, Rev: 5’-AATGATATCTCACTTGTACAGCTCGTCCATGC-3’), and cloned into the GFP-null *RECQL4* vector, resulting in the expression of a GFP fusion at the C-terminus of RECQL4.

The two mutant versions of RECQL4 (with either the substitution of P532S or R766Q) were generated by site-directed mutagenesis performed on the wild type *RECQL4* with the suitable primer pairs. The following primers were used: RECQL4_P532S_: Fwd: 5’- CCTCACGTTGGTCGTCTCTTCCCTGCTGTC-3’, Rev: 5’-GACAGCAGGGAAGAGACGACCAACGTGAGG-3’; RECQL4_R766Q_: Fwd: 5’-AGGGCCAGTTGCAGGTGGTGGTGGC -3’, Rev: 5’- GCCACCACCACCTGCAACTGGCCCT -3’. All resulting sequences were confirmed by Sanger sequencing.

### Plasmid DNA isolation and cell transfection

DNA preparations of all RECQL4 plasmids (wildtype and two mutants, N- and C-GFP tagged versions) were isolated using Qiagen Mini Prep and Qiagen Midi Prep kits, following the manufacturer’s protocol. Transfection of glioma cells with specific plasmids was carried out using Lipofectamine™2000 transfection reagents, according to the manufacturer’s protocol. Briefly, LN18 and LN229 cells were trypsinized and 2 × 10^5^ cells were seeded in 6-wells plates 24 h before transfection. Directly prior to transfection, the cells were washed twice in pre-warmed PBS and incubated in antibiotic-free medium. Plasmids: RQ4-WT, RQ4-M1, RQ4-M2 (0.5 µg/each) were diluted in 100 µL Opti-MEM™ medium, then mixed with 100 µL Lipofectamine™2000 transfection reagent. After 20 min, the mix was added to the cells and incubated for 6 h at 37 °C. The medium was exchanged with regular medium 6 h after transfection.

### RECQL4 immunoprecipitation

For GFP-trap experiments, cells expressing GFP fusion proteins were lysed in a modified RIPA buffer. For one immunoprecipitation reaction, we used ~10^6^-10^7^ cells. Cell pellet was suspended in 200 μL ice-cold RIPA buffer (50 mM Tris/HCl pH 7.4; 150 mM NaCl; 20 mM NaF; 1 mM EDTA 1% sodium deoxycholate, 2.5 mM sodium pyrophosphate, 1 mM beta-glycerophosphate, 1 mM Na_3_VO_4)_, supplemented with DNaseI (75-150 Kunitz U/mL), MgCl_2_ (2.5 mM), the protease inhibitor mix (5 µg Aprotinin and Leupeptin) and 1 mM phenylmethylsulfonyl fluoride (PMSF). After incubation on ice for 20 min, lysates were centrifuged at 14,000 × g for 15 min, and the supernatant was used as total cell extract. The suspension was placed on ice for 30 min, with pipetting every 10 min. cell lysate were centrifugated at 17,000x g for 10 min at +4 °C. Cleared lysate was transferred to a fresh tube and 300 μL Dilution buffer supplemented with 1 mM PMSF and protease inhibitor cocktail was added. Beads (25 μL) were gently pipetted in 500 μL ice-cold Dilution buffer, and separated with a magnet (this step was repeated twice). Beads were equilibrated 3x with 500 ul μL ice-cold Dilution buffer. Aliquots of cell extracts were incubated with GFP-trap beads overnight at 4 °C. The beads were washed 4 times with RIPA buffer, separated with a magnet, and resuspended in 500 μL Wash buffer (this step was repeated twice). Beads were resuspended in 80 μL 2x SDS-sample buffer and boiled for 5 min at 95 °C to dissociate immunocomplexes from beads. The beads were separated with a magnet, the supernatant collected and. Bound proteins and input samples were analyzed by SDS-PAGE/Western blotting with appropriate antibodies, followed by HRP-conjugated secondary antibodies. Immunoreactive protein bands were detected by chemiluminescence (Bio-Rad).

Immunoprecipitation of RQ4-WT, RQ4-M1, RQ4-M2-GFP coupled proteins from transfected LN18 and LN229 cells was performed using Chromotek GFP-Trap magnetic agarose beads (gtma-20, Chromotec, München) according to the manufacturer’s protocol.

### Cell viability and proliferation

Cells were seeded at a density of 4 × 10^3^ cells/well and cell viability was determined using PrestoBlue™ reagent according to manufacturer’s recommendation. Briefly, RECQL4 deficient glioma cells were transfected with plasmids carrying the wildtype RECQL4 or its mutant versions. For drug treatments, cells were exposed 24 h after transfection to the compounds and incubated for an additional 48 h. On the day of cell harvesting, the medium was replaced with fresh containing PrestoBlue™ reagent at 1:10 ratio. Following 30 min incubation, 100 µL of the PrestoBlue™ medium was transferred into 96-well plate and absorbance at wavelength of 570 nm and 620 nm was measured using a scanning multi-well spectrophotometer. The remaining cells were harvested for total protein extraction and Western blotting analysis. Cell viability measurements were normalized to the protein content in a given sample. Cell proliferation was determined using ELISA BrdU kit (Roche Diagnostics GmbH, Germany) according to the manufacturer’s protocol.

### Western blot analysis

The total protein extracts were resolved on 8% or 10% SDS-PAGE gels (Eurogentec) and transferred onto nitrocellulose membranes (Bio-Rad). The proteins were detected with anti-TAP (Thermo Fisher), anti-MYC (Cell Signaling), or anti-Flag (Sigma) antibodies. Immunocomplexes were detected using SuperSignal West Pico^PLUS^ Chemiluminescent Substrate (Thermofisher Scientific, Rockford, IL, USA) and visualized by ChemiDoc Imaging System (Bio-Rad Laboratories, Hercules, CA, USA). GAPDH or α-tubulin antibodies were used as control for equal protein loading. Membranes were stripped in 100 mM glycine and 20% SDS buffer, pH 3.0 for 30 min at RT, washed, blocked, and re-probed with antibodies (shown in supplementary Table 1). Densitometric analyses were performed using Image Lab ver. 5.2 software (Bio-Rad Laboratories, Hercules, CA, USA).

### Immunofluorescence

Approximately 3.5 × 10^5^ cells were seeded on 12 mm coverslips, and 24 h later, cells were transfected with plasmid containing wildtype or mutant RECQL4-GFP tagged constructs. After an additional 24 h, cells were exposed to 30 J/m^2^ dose of UV-C in the Transilluminator and left for 24 h. Cells were washed three times with 1 × PBS and fixed with 4% PFA in 1 × PBS for 10 min at RT. Cells were then washed three times for 5 min each with 1 × PBS, stained with respective antibodies recognizing BLM or γH2AX, and after washing with 3 × PBS, the nuclei were counterstained with 0.2 µg/mL DAPI in 1 × PBS, and then washed for 5 min with water before examination of fluorescence. The coverslips were mounted on slides with VectaShield mounting medium (Vector Laboratories) and examined with an epifluorescent microscope (BX51, Olympus). A total of approximately 200 GFP-expressing cells with either WT or RECQL4 variants were analyzed from three different experiments.

### Statistical analysis

All biological experiments were performed on independent 3-4 cell passages. Results are expressed as means ± standard deviation (SD). P values were calculated using chi-square test, two-tailed t-test, one-way or two-way ANOVA followed by appropriate post-hoc test using GraphPad Prism v6 (GraphPad Software, San Diego, CA, USA).

## Supplementary information

Supplementary figure S1

Supplementary figure S2

Uncropped blots for figs.2, 4

Supplementary table S1

## Data Availability

The RNAseq datasets generated and analyzed in the current study are available in the repository. Other materials are available from the corresponding authors on reasonable request.
