# Supplementary material for: Novel mutations in the RECQL4 gene affect its helicase functions, interactions with the BLM helicase and chemotherapeutics-induced cell death
Source: Cell Death Discov. 2025 Dec 19;11:560. doi: 10.1038/s41420-025-02834-w (PMC12717039; doi:10.1038/s41420-025-02834-w)
Supplement: Supplementary file 1 — Supplementary figure S1 [file 41420_2025_2834_MOESM1_ESM.pdf]

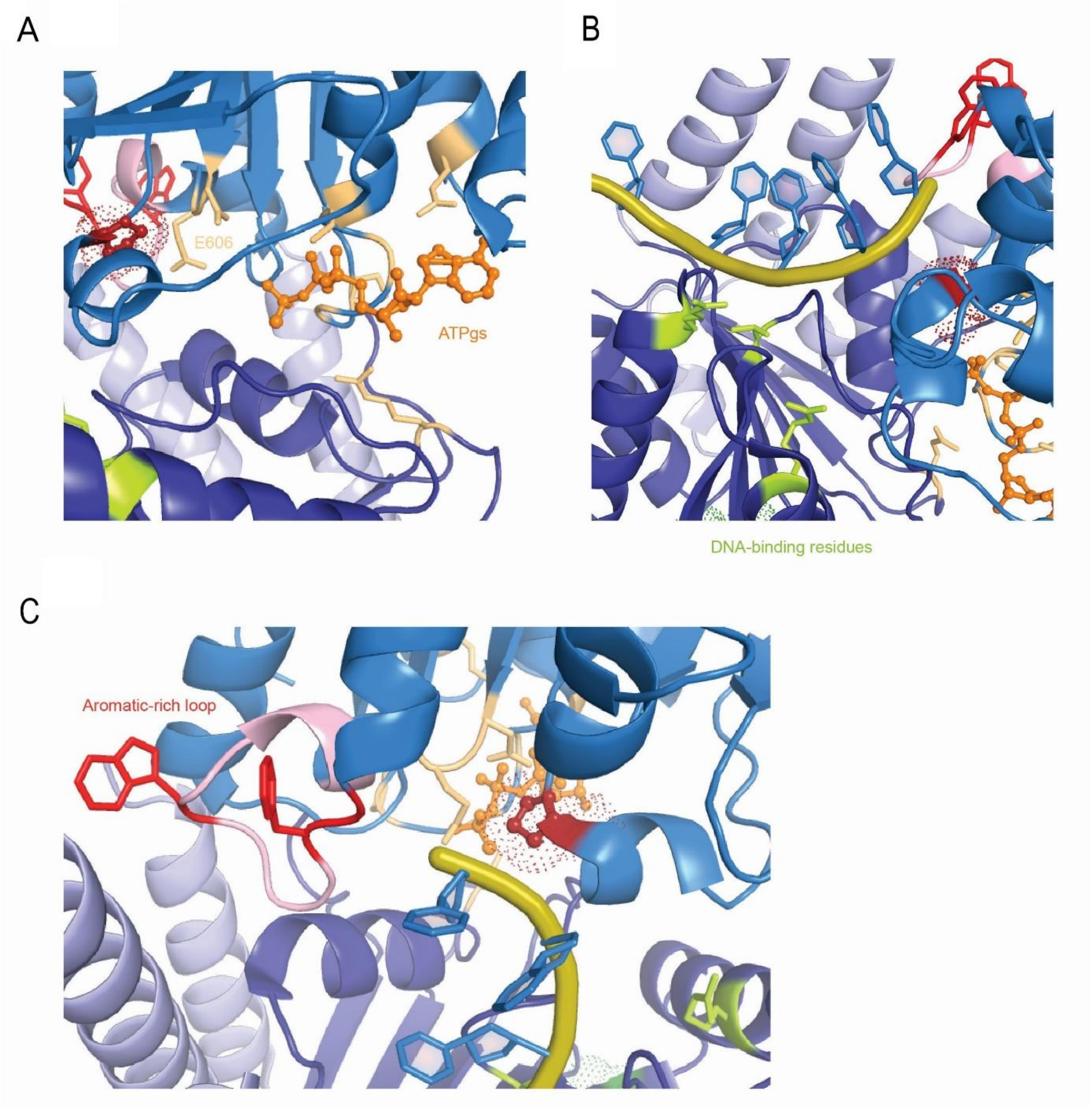

Supplementary figure S1. Modelling RECQL4 and DNA interactions. A. The modelled ATP molecule in RECQL4 is expected to accurately represent the native binding site of nucleoside triphosphates. The position of the DNA in the RECQL4$_{427\text{-}1116}$ structures is marked. B. Many conserved residues, that are involved in single stranded DNA binding, fall proximal to the modelled DNA. C. Residues within the aromatic-rich loop (ARL) are proximal to the 3’ end of the ssDNA**.**
