# Supplementary material for: Novel mutations in the RECQL4 gene affect its helicase functions, interactions with the BLM helicase and chemotherapeutics-induced cell death
Source: Cell Death Discov. 2025 Dec 19;11:560. doi: 10.1038/s41420-025-02834-w (PMC12717039; doi:10.1038/s41420-025-02834-w)
Supplement: Supplementary file 2 — Supplementary figure S2 [file 41420_2025_2834_MOESM2_ESM.pdf]

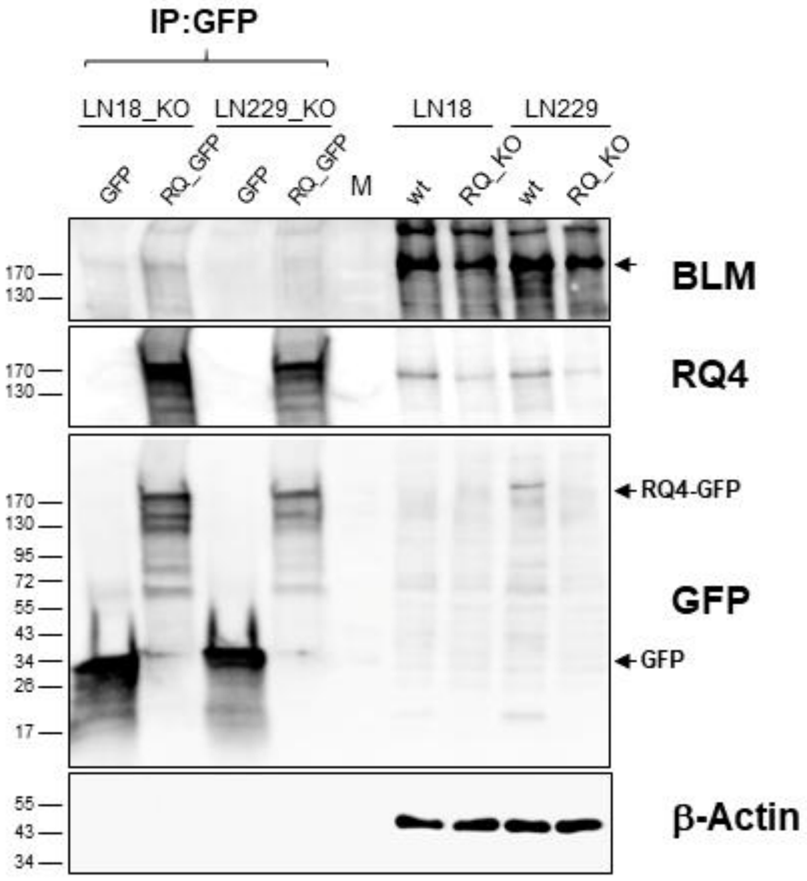

Supplementary fig. S2. Studies of RECQL4 variants and BLM interactions in glioma cells. RQ4wt was overexpressed in RQ4 KO LN18 and LN229 cells, and after 24 h the complexes of RECQL4-GFP with interacting proteins were immunoprecipitated (IP) with an antibody against GFP. The cells transfected with GFP alone did not show a non-specific signal with the BLM or RECQL4 antibodies after immunoprecipitation.
