# Supplementary figures and images for: Novel mutations in the RECQL4 gene affect its helicase functions, interactions with the BLM helicase and chemotherapeutics-induced cell death
Source: Cell Death Discov. 2025 Dec 19;11:560. doi: 10.1038/s41420-025-02834-w (PMC12717039; doi:10.1038/s41420-025-02834-w)

LN18

LN229

GFP

GFP

Tubulin

LN229

LN18

PARP

RECQL4

Tubulin

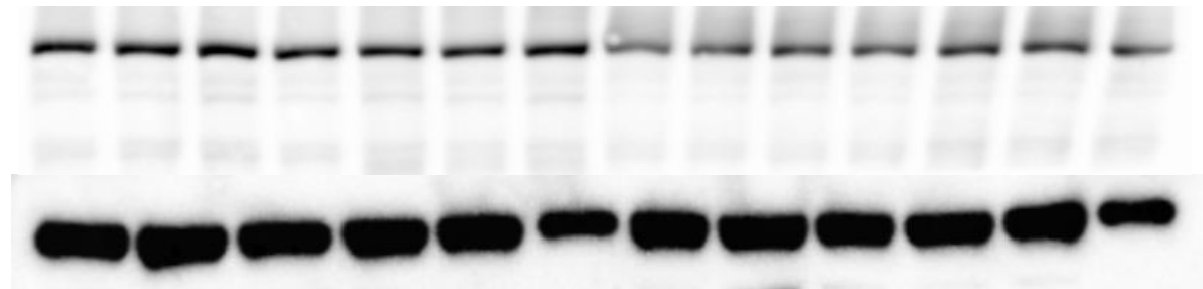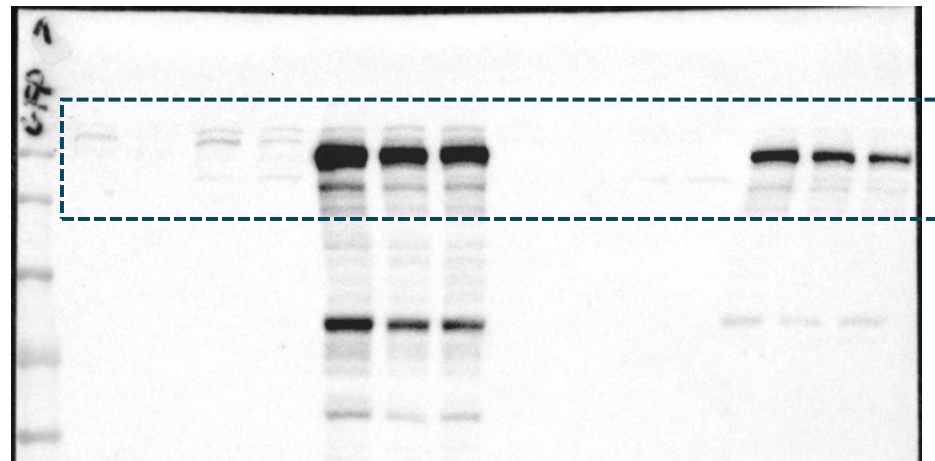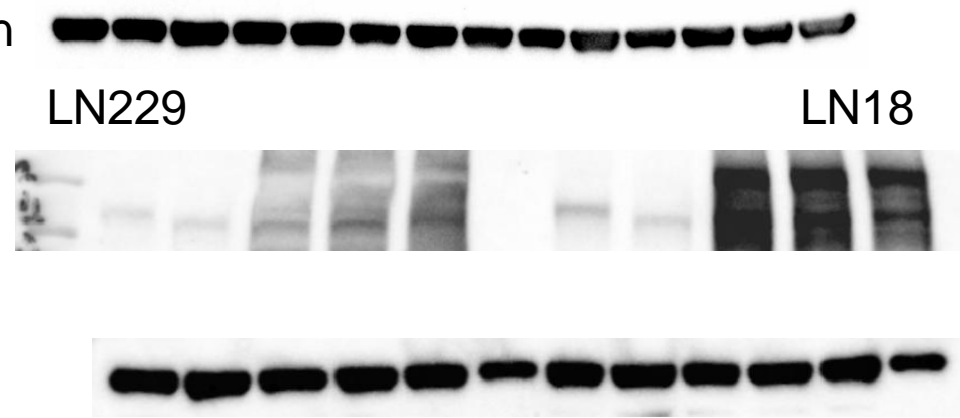

Fig. 4

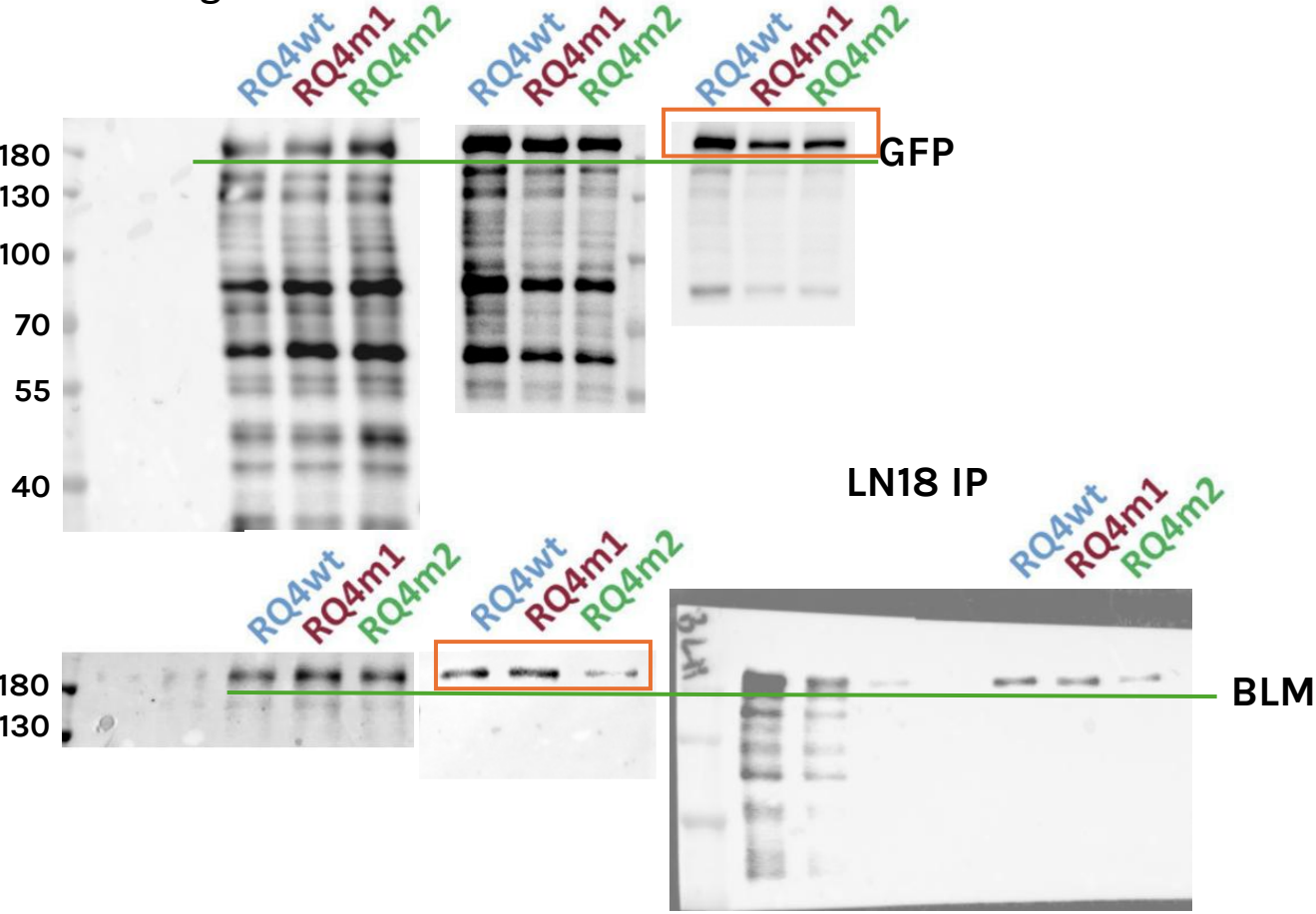

Fig.S2

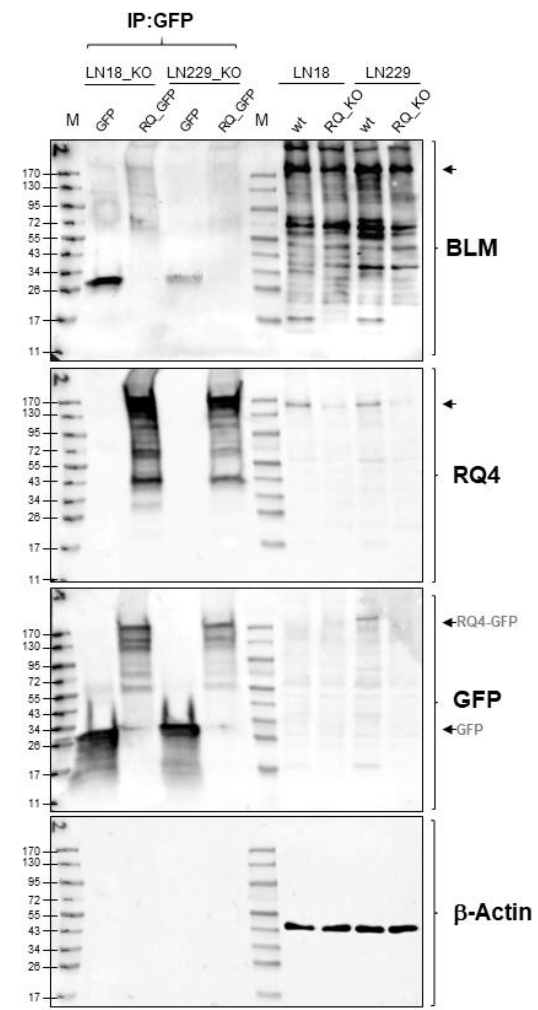

Supplement: Supplementary file 3 — Uncropped blots for figs.2, 4 [file 41420_2025_2834_MOESM3_ESM.pdf]
