# Supplementary material for: Novel mutations in the RECQL4 gene affect its helicase functions, interactions with the BLM helicase and chemotherapeutics-induced cell death
Source: Cell Death Discov. 2025 Dec 19;11:560. doi: 10.1038/s41420-025-02834-w (PMC12717039; doi:10.1038/s41420-025-02834-w)
Supplement: Supplementary file 4 — Supplementary table S1 [file 41420_2025_2834_MOESM4_ESM.pdf]

Supplementary table S1. A list of antibodies used in the study

Antibody	Manufacturer	Cat. number	Dilution
GFP	Santa Cruz	sc-8334	1:1000
RECQL4	Cell Signaling<br>Novus Biologicals	2814<br>25470002	1:1000<br>1:1000
BLM	Sigma Aldrich<br>Abcam	HPA005689<br>ab2179	1:1000<br>1:1000
PARP	Cell Signaling	9542	1:1000
CyclinB1	Santa Cruz	sc-245	1:1000
phospho-CHK1	Cell Signaling	9947	1:1000
phospho-H2AX	Cell Signaling	9718	1:1000
cleaved caspase 7	Cell Signaling	9491	1:1000
α-tubulin (DM1A)	Santa Cruz	sc-32293	1:1000
Lamin B1	Santa Cruz	sc-365962	1:500
HNK1	Developmental Studies Hybridoma Bank (DSHB)	zn-12	1:500
Isl1	Developmental Studies Hybridoma Bank (DSHB)	39.4D5	1:500
